# Sphere-Forming Culture for Expanding Genetically Distinct Patient-Derived Glioma Stem Cells by Cellular Growth Rate Screening

**DOI:** 10.3390/cancers12030549

**Published:** 2020-02-27

**Authors:** Kayoung Shin, Hyemi Shin, Hee Jin Cho, Hyunju Kang, Jin-Ku Lee, Yun Jee Seo, Yong Jae Shin, Donggeon Kim, Harim Koo, Doo-Sik Kong, Ho Jun Seol, Jung-Il Lee, Hye Won Lee, Do-Hyun Nam

**Affiliations:** 1 Department of Health Sciences and Technology, Samsung Advanced Institute for Health Science and Technology, Sungkyunkwan University, Seoul 06531, Korea; skyoung92@g.skku.edu (K.S.); guhalim@g.skku.edu (H.K.); 2 Research Institute for Future Medicine, Samsung Medical Center, Seoul 06351, Korea; shinhm0427@g.skku.edu (H.S.); heejin1017.cho@samsung.com (H.J.C.); yunjee.seo@aimedbio.com (Y.J.S.); yongjae.shin@samsung.com (Y.J.S.); dg.kim@g.skku.edu (D.K.); 3 Precision Medicine Research Institute, Samsung Medical Center, Seoul 06351, Korea; 4 Graduate School of Biomedical Science, Ajou University School of Medicine, Suwon 16499, Korea; hjkang@yonsei.ac.kr (H.K.); jinkulee@ajou.ac.kr (J.-K.L.); 5 Department of Neurosurgery, Samsung Medical Center, Sungkyunkwan University School of Medicine, Seoul 06531, Korea; kds026@skku.edu (D.-S.K.); hojun.seol@samsung.com (H.J.S.); jilee@skku.edu (J.-I.L.); 6 Department of Hospital Medicine, Yonsei University College of Medicine, Seoul 03722, Korea

**Keywords:** diffuse infiltrating glioma, personalized medicine, glioma stem cells, short-term cultivation screening platform, growth factor, genomic profiling

## Abstract

Diffusely infiltrating gliomas (DIGs) are difficult to completely resect and are associated with a high rate of tumor relapse and progression from low- to high-grade glioma. In particular, optimized short-term culture-enriching patient-derived glioma stem cells (GSCs) are essential for customizing the therapeutic strategy based on clinically feasible in vitro drug screening for a wide range of DIGs, owing to the high inter-tumoral heterogeneity. Herein, we constructed a novel high-throughput culture condition screening platform called ‘GFSCAN’, which evaluated the cellular growth rates of GSCs for each DIG sample in 132 serum-free combinations, using 13 previously reported growth factors closely associated with glioma aggressiveness. In total, 72 patient-derived GSCs with available genomic profiles were tested in GFSCAN to explore the association between cellular growth rates in specific growth factor combinations and genomic/molecular backgrounds, including isocitrate dehydrogenase 1 (*IDH1*) mutation, chromosome arm 1p and 19q co-deletion, ATRX chromatin remodeler alteration, and transcriptional subtype. GSCs were clustered according to the dependency on epidermal growth factor and basic fibroblast growth factor (E&F), and isocitrate dehydrogenase 1 (*IDH1*) wild-type GSCs showed higher E&F dependencies than *IDH1* mutant GSCs. More importantly, we elucidated optimal combinations for *IDH1* mutant glioblastoma and lower grade glioma GSCs with low dependencies on E&F, which could be an aid in clinical decision-making for these DIGs. Thus, we demonstrated the utility of GFSCAN in personalizing in vitro cultivation to nominate personalized therapeutic options, in a clinically relevant time frame, for individual DIG patients, where standard clinical options have been exhausted.

## 1. Introduction

Diffusely infiltrating gliomas (DIGs) are categorized by the World Health Organization (WHO) into diffuse astrocytomas, oligodendrogliomas, and isocitrate dehydrogenase 1 and 2 (*IDH1*/*2*) mutant (mut) and wild-type (wt) glioblastomas, according to their histological grades, *IDH1/2* status, and chromosome arm 1p and 19q (1p19q) co-deletion [1,2]. DIGs are among the most devastating tumor types and are characterized by extensive infiltrative growth, a low probability of complete resection, and resistance to combined therapies [3,4]. Glioblastoma is a WHO grade IV disease with poor prognosis, and only about 10% of these malignancies possess *IDH1/2* mutation without 1p19q co-deletion [2]. On the other hand, diffuse astrocytomas (*IDH* mutant and 1p19q intact) and oligodendrogliomas (*IDH* mutant and 1p19q co-deleted) belong to WHO grades II and III, which represent "lower-grade gliomas" (LGGs) in The Cancer Genome Atlas (TCGA) [1,2,3,4]. Most importantly, LGGs not only recur over time, but also progress to high-grade gliomas (HGGs) that are characterized by the high inter- and intra-tumoral heterogeneity associated with rapid clinical deterioration [3,4,5].

Recently, the three-dimensional (3D) organoid culture system has been reported to recapitulate the biology of parental tumors and their microenvironment in glioblastomas [6,7]. Organoid culture methods have definite advantages in mimicking the in vivo microenvironment and biological complexities of original tumors [8], but the two-dimensional (2D) spheroid culture system is still preferred as it is more reproducible, flexible and suitable for elucidating cancer drug responses and, in particular, the response of glioma stem cells (GSCs) with a high-throughput large scale [9,10,11] compared to other 3D organotypic spheroids [12,13,14,15,16]. As tumor evolution and inevitable recurrence after therapy are likely attributed to GSCs [17], short-term cultured patient-derived GSCs could facilitate real-time precision treatment decisions via high-throughput drug screening assays, and would, therefore, be reliable tools for translational research. Thus, a variety of efforts have been made to enrich GSCs from patient tumor tissues while maintaining the heterogeneity of mature tumors and retaining their major genomic/molecular characteristics [14,16,18]. 

As GSCs share similarities with neural stem cells (NSCs), the neural basal medium supplemented with epidermal growth factor (EGF) and basic fibroblast growth factor (bFGF) (E&F; referred to as NBE) has been widely used for GSCs [14,16,18,19,20]. However, GSCs are distinguished from NSCs by their chromosomal alterations, cytologic and nuclear atypia, aberrant differentiation, failure to respond to growth inhibitory cues and tumor growth [19]. For example, NBE is effective only for GSCs in patients with *IDH/2* wt glioblastomas [14,18]. Although informing precision treatment decisions against *IDH1*-mutated (mut) glioblastomas and LGGs has been limited by the lack of clinically relevant translational models, some of them have become refractory to standard therapies after recurrence [3,4,5,21], therefore, a short-term culture system providing appropriate strategies within the time window of the first therapy is urgently needed. 

**Figure 1:**
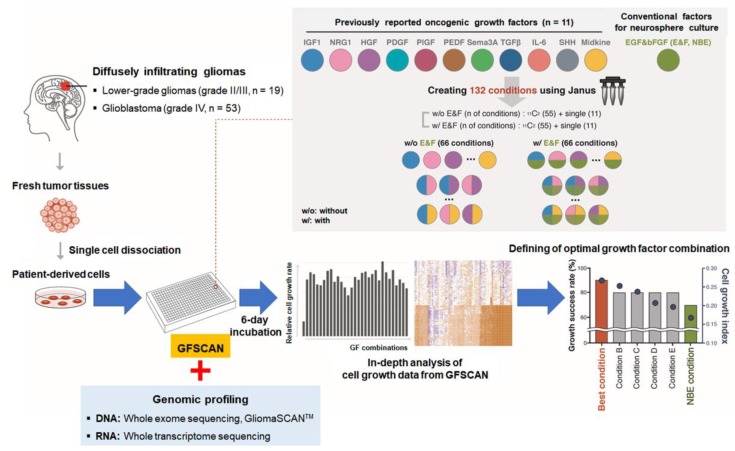
Schematic workflow of the Growth Factor SCAN (GFSCAN) to identify optimal growth factor conditions for DIGs. For 19 LGG and 53 glioblastoma GSCs, GFSCAN was performed to evaluate the effects of 132 growth factor combinations on cell growth, and their genomic characteristics were determined by next-generation sequencing.

Hence, to personalize in vitro culture conditions for GSCs derived from individual patients with distinct genomic traits, we constructed a cell growth screening platform, named ‘Growth Factor SCAN (GFSCAN).’ GFSCAN provides 132 distinct culture conditions generated by 11 oncogenic growth factors (GFs) and E&F combinations in a 384-well high-throughput screening format in a fast and cost-effective manner (Figure 1 and Table S1). In this study, GFSCAN and next-generation sequencing (NGS) were performed on 53 glioblastoma and 19 LGG stem cells from 60 patients, including multi-sector samples (Table S2). The obtained GFSCAN and genomic data were combined to determine the best culture conditions according to *IDH1* mutations, 1p19q co-deletion, and ATRX chromatin remodeler *(ATRX)* gene mutations, which are key alterations in DIG (Figure 1).

## 2. Results

### 2.1. Reproducibility and Robustness of the GFSCAN Platform

The biological stability of each GF and cell doubling time should be considered when evaluating the ability of GFs to promote in vitro cell growth. As each GSC shows variable doubling times ranging from 2 to 9 days, we determined the GFSCAN screening period to be 6 days, so that cells could grow without media addition (Figure 1). To measure cell proliferation rates under each culture condition, we defined a new cell growth index as (D6 - N6)/D0, where D0, D6, and N6 indicate the quantitative GFSCAN readout on the day of initial cell seeding (D0), after 6 days of incubation under each culture condition (D6), and after 6 days of incubation in basal media (N6), respectively (Figure 1). The cell growth index represented cell growth 6 days after initial cell seeding and also the effects of growth factors on cell growth compared to cell viability in the basal media without GFs. We screened two cases (BT-S07T and BT-S08T) on GFSCAN plates by seeding dissociated cells from the same patient, thawed from two independently frozen vials. The cell growth indices under each condition were highly correlated in both cases (R^2^ = 0.9957 and 0.9898, respectively) (Figure S1). Furthermore, GSCs from LGGs and glioblastomas showed distinct transcriptome profiles (Figure S2A,B) and the cell growth rates (D6/D0) under the NBE condition of glioblastoma GSCs were significantly higher compared to those of LGG GSCs (*p* = 0.0027) (Figure S2C), which was consistent with the up-regulated cell cycle activity in glioblastomas compared to that of LGGs (Figure S2D), indicating the reproducibility and robustness of our GFSCAN platform.

### 2.2. Genomic Profiles of 72 DIGs in the GFSCAN Cohort

**Figure 2:**
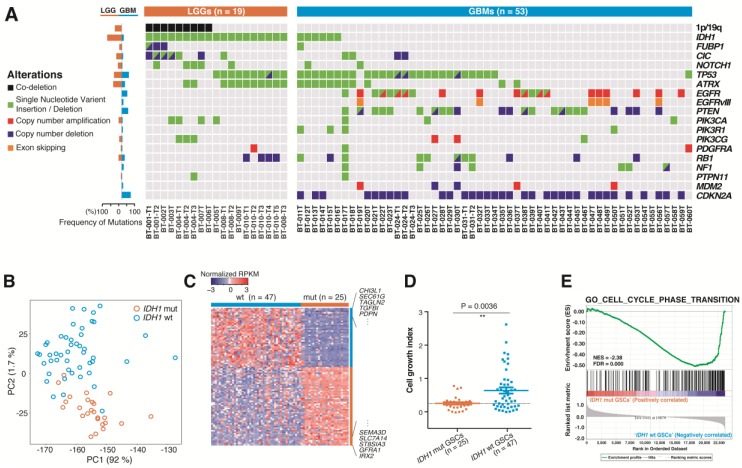
Genomic landscape of 72 diffusely infiltrating gliomas (DIGs) and distinct genomic characteristics between *IDH1*-mutated (mut) and wild-type (wt) glioma stem cells (GSCs). (**A**) The alterations in the frequently mutated genes in DIGs for 19 lower-grade gliomas (LGGs) and 53 glioblastomas. On the left, the alteration frequency of each gene was compared between LGGs and glioblastomas. (**B**) The principle component analysis (PCA) of the gene expression profiles of 72 DIGs (*IDH1*-wt (n = 47) and *IDH-*mut DIGs (n = 25), using newly curated tumor-intrinsic genes (refer to Materials and Methods). The blue and orange dots indicate *IDH1*-wt and *IDH1*-mut GSCs, respectively. (**C**) Differentially expressed genes between GFSCAN *IDH1*-wt and -mut GSCs (*p* value ≤ 0.05 and Benjamini q value ≤ 0.1). (**D**) The cell growth index of *IDH1*-mut and -wt GSCs in neural basal medium supplemented with EGF and bFGF (NBE) (*p* = 0.0027). (**E**) The gene-set enrichment analysis (GSEA) plot of a cell cycle-associated pathway (GO_CELL_CYCLE_PHASE_TRANSITION) between *IDH1*-mut and -wt GSCs.

To determine the genomic and expression profiles of 72 DIGs, tissues or cultured GSCs were subjected to whole exome sequencing (WES), glioma-specific targeted DNA sequencing (GliomaSCAN^TM^), and whole transcriptome sequencing (WTS) (Tables S2 and S3). Recently performed large-scale studies reported that the genomic profiles of LGGs and glioblastomas are distinct; glioblastomas show frequent alterations in the epidermal growth factor receptor (*EGFR*), phosphatase and tensin homolog (*PTEN*), and cyclin-dependent kinase inhibitor 2A (*CDKN2A*), whereas genes encoding *IDH1/2*, *ATRX*, tumor protein *P53* (*TP53*), capicua transcriptional repressor (*CIC*), and far upstream element binding protein 1 (*FUBP1*) are frequently altered in LGGs with or without 1p19q co-deletion [1,3,4,22]. We assessed genomic alterations in these previously reported genes for the 72 DIGs subjected to GFSCAN (Figure 2A and Table S3). In our cohort, all LGGs (n = 19) and six out of 53 (11.3%) of the glioblastomas harbored *IDH1* mutations; among the LGGs, nine (47.4%) showed 1p19q co-deletion, while no samples showed 1p19q co-deletion in glioblastomas. 

Other frequently mutated genes in glioblastomas, such as *EGFR* and *PTEN*, displayed similar mutation frequencies as in previous reports [1,3,4,22], although *IDH1*, *TP53*, and *ATRX* mutations occur more frequently in this glioblastoma cohort (Figure 2A and Table S3). Additionally, *FUBP1*, *CIC*, Notch homolog 1, translocation-associated (Drosophila) (*NOTCH1)*, *ATRX*, and *TP53* mutations were accompanied by *IDH1* mutation in GFSCAN LGGs (Figure 2A and Table S3), which is consistent with the results of previous studies [1,3,4]. To summarize, the DIG samples used in the present study showed similar genomic characteristics as an external dataset analyzed in recent studies, indicating that our cohort was relevant and representative, despite the small number of cases.

### 2.3. Molecular and Biological Characteristics of Patient-Derived GSCs with IDH1 Mutation

In general, *IDH1* mutation is a critical biomarker that is used to classify DIGs into clinically and biologically similar subgroups [3,4]. Mutant *IDH* is frequent in LGGs but also detected in 50–88% of secondary glioblastomas and in 5% of primary glioblastomas, resulting in altered 2-hydroxyglutarate production and altered DNA methylation [4,22,23]. More importantly, *IDH1*-mut GSCs are difficult to propagate in vitro and even in orthotopic xenografts compared to *IDH1*-wt GSCs [24,25,26]. As expected, GSCs from *IDH1*-wt (n = 47) and -mut DIGs (n = 25) showed remarkable differences in their gene expression profiles (Figure 2B,C), and *IDH1*-wt GSCs showed significantly higher cell growth indices under conventional NBE conditions (*p* = 0.0036; Figure 2D), in addition to more dysregulated cell proliferation signatures (Figure 2E). 

**Figure 3:**
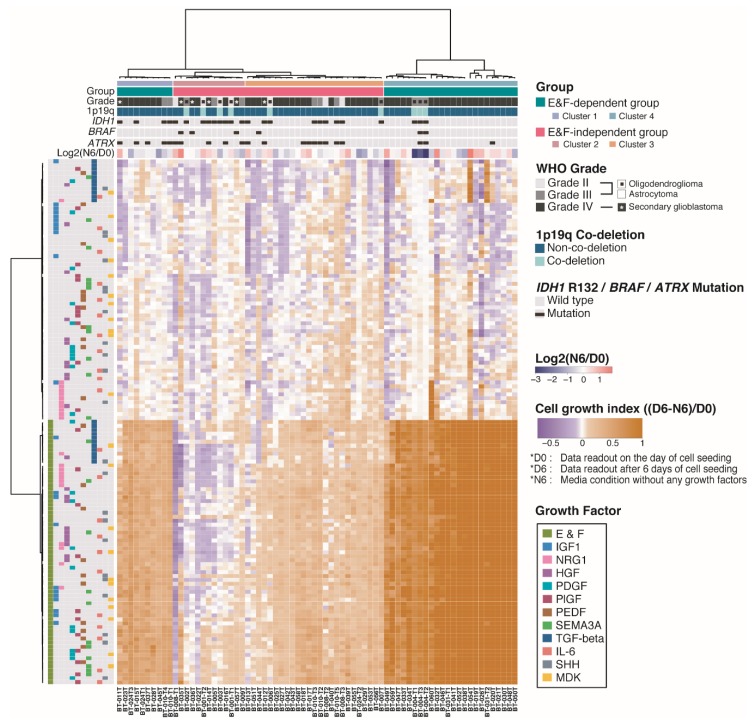
Unsupervised clustering of cell growth indices evaluated by GFSCAN in 72 DIGs. The unsupervised clustering revealed that DIGs are categorized according to their E&F dependencies.

To optimize culture media conditions for GSCs that did not show adequate cell growth under NBE conditions, all GSCs were first clustered according to their cell growth indices in GFSCAN, resulting in four subgroups (Figure 3 and Table S4). GSCs in Clusters 1 and 4 showed increased cell growth indices in the presence of E&F compared to E&F-absent conditions (E&F-dependent), while GSCs in Clusters 2 and 3 were less affected by E&F (E&F-independent). Most GSCs from LGGs were included in the E&F-independent group (E&F-independent; n = 14, 73.7% vs. E&F-dependent; n = 5 26.3%) and, consistently, 72% of *IDH1*-mut GSCs were present in the E&F-independent group. 

### 2.4. Pigment Epithelium-Derived Factor (PEDF)/Midkine (MDK)/E&F-Containing Media Promoted In Vitro GSC Propagation from IDH1-wt Glioblastomas 

**Figure 4:**
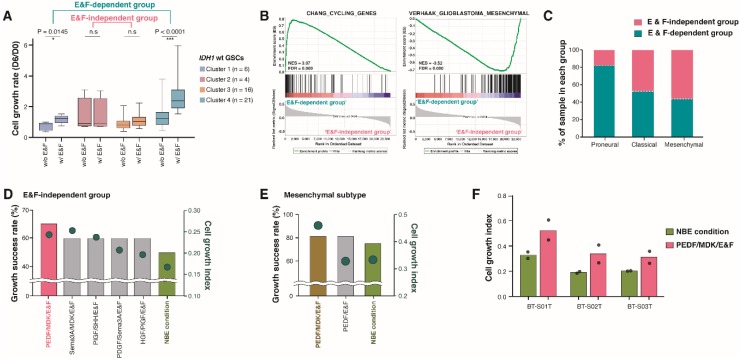
Identification of new culture conditions in E&F-independent GSCs among *IDH1*-wt GSCs. (**A**) The 6-day cell proliferation rates of each cluster without or with E&F. The E&F-dependent group differed significantly in 6-day cell growth rates according to whether E&F were present (Clusters 1 and 4, *p* = 0.0145 and *p* < 0.0001, respectively), while E&F-independent 6-day cell growth rates were not affected by the absence of E&F (Clusters 2 and 3). (**B**) The enrichment of a cell cycle-associated gene set (KONG_E2F3_TARGETS, the left panel) in E&F-dependent group and enrichment of a glioblastoma mesenchymal gene set (VERHAAK_GLIOBLASTOMA_MESENCHYMAL, the right panel) in the E&F-independent group. (**C**) The distribution of glioblastoma subtypes into independent and dependent groups in *IDH1*-wt DIGs. (**D**–**E**) The culture conditions with higher growth success rates than the NBE condition for E&F-independent group (**D**) and mesenchymal *IDH1*-wt GSCs (**E**). The bars and dots indicate growth success rates and cell growth indices, respectively. (**F**) The cell growth indices of *IDH1-*wt GSCs from a validation cohort under epidermal growth factor (EGF) and basic fibroblast growth factor (bFGF) (E&F; referred to as NBE) and pigment epithelium-derived factor (PEDF)/midkine (MDK)/E&F conditions. The dots represent the values from technical duplicates.

*IDH1*-wt GSCs showed higher growth indices in NBE than *IDH1*-mut GSCs (Figure 2D), but not all GSCs from *IDH1*-wt glioblastomas were dependent on E&F for cell growth (Figure 3 and Figure 4A). For example, all four *IDH1*-wt glioblastoma GSCs with *BRAF* hotspot mutations (V600E and D594H) were classified into the E&F-independent group (Figure 3 and Table S4), although *BRAF* alterations are rarely observed in adult HGG or other diffusely infiltrating gliomas (2–5%) [22]. 

Interestingly, gene-set enrichment analysis (GSEA) revealed that genes related to cell proliferation were enriched in E&F-dependent *IDH1*-wt glioblastoma GSCs, while genes related to a mesenchymal subtype signature [27] were enriched in the E&F-independent group (Figure 4B). Similarly, when we determined the tumor-intrinsic molecular subtypes of *IDH1*-wt GSCs based on gene expression profiles [28], *IDH1*-wt GSCs with a proneural subtype (n = 11) were more prevalent in the E&F-dependent group (10% (2/20) in E&F-independent vs. 34.6% (9/26) in E&F-dependent), while the tumor-intrinsic mesenchymal subtype (n = 16) was more dominant in the E&F-independent group (45% (9/20) in E&F-independent vs. 26.9% (7/26) in E&F-dependent) (Figure 4C). The tumor-intrinsic classical subtype (n = 19) occurred in the independent and dependent groups at comparable rates (45% (9/20) in E&F-independent vs. 38% (10/26) in E&F-dependent). 

Based on these results, we determined the most efficient GF combination for the expansion of *IDH1*-wt GSCs with mesenchymal traits that were closely associated with the most aggressive DIG subset [28]. First, we calculated the growth success rate for each condition, where growth success rate was defined as the ratio of the number of GSCs with D6/D0 > 1 to the number of total GSCs. *IDH1*-wt GSCs within the E&F-independent group showed the highest growth success rate under PEDF/MDK/E&F conditions (Figure 4D), which was identical to the most optimal growth factor composition for cell growth in the mesenchymal subtype (Figure 4E). Previously, MDK has been reported as a secretion protein that promotes glioma cell proliferation [29]. We evaluated the effects of an MDK-neutralizing antibody on an E&F-independent GSC from our GFSCAN cohort (BT-030T) using gene expression profiling, and cell cycle and proliferation-associated gene sets were significantly down-regulated in the MDK-neutralizing antibody-treated sample compared to the control sample (Figure S3). 

To verify our findings in independent *IDH1*-wt glioblastoma cohorts, we first obtained the differentially expressed genes (DEGs) between E&F-dependent and -independent *IDH1*-wt GSCs in the GFSCAN cohort (Table S5), and then we defined the DEGs as surrogate markers to classify *IDH1*-wt glioblastomas with gene expression profiles into either the E&F-dependent or -independent groups. Using the defined surrogate markers, we categorized an additional 202 in-house *IDH1*-wt glioblastomas into E&F-dependent or -independent samples that were not included in the screening set (Figure S4A). Consistent with the original screening dataset, the mesenchymal subtype and *BRAF* mutations were dominant in the E&F-independent group, and over 90% of proneural glioblastomas belonged to the E&F-dependent group (Figure S4B,C). Among the E&F-independent samples, we selected three *IDH1*-wt GSCs (BT-S01T and BT-S02T: mesenchymal subtype; BT-S03T: classical subtype) and evaluated their growth rates with GFSCAN. This confirmed that the cell growth indexes of the three GSCs were 1.6-fold, 1.8-, and 1.5-fold higher, respectively, under PEDF/MDK/E&F conditions than under the NBE condition, as expected (Figure 4F). 

### 2.5. Newly Identified Conditions for the Cultivation of IDH1-mut GSCs 

It has been reported that patient-derived GSCs with IDH1 mutation were hardly grown in conventional culture conditions and scarcely established in preclinical platforms previously [24,26,30,31,32]. *IDH1/2* mutations have been estimated to occur in 54–100% of diffuse astrocytomas (WHO II), 66.1% of anaplastic astrocytomas (WHO III), and 64–93% of oligodendrogliomas (WHO II and III) cases [22]. In addition, 1p19q co-deletion is a genetic marker that can distinguish oligodendroglioma from astrocytoma [1,3,4,22]. LGGs with an *IDH1* mutation and 1p19q co-deletion show oligodendroglioma histological characteristics and arise from *TERT* activation, mutations in *CIC* and *FUBP1*, and activating alterations in the phosphoinositide-3-kinase (PI3K) pathway [1,3,4,22]. On the other hand, LGGs with *IDH1* mutation and no 1p19q co-deletion accompanied by *ATRX* and *TP53* mutations represent astrocytomas. Unsupervised clustering using the cell growth index from GFSCAN revealed that *IDH1*-mut GSCs were dominantly classified into the E&F-independent group (Figure 3, *p* = 0.0253) consistent with previous findings [10,24,25,26]. Therefore, it is important to define individualized culture conditions for cultivating *IDH1*-mut GSCs according to the presence of these major alterations. 

**Figure 5:**
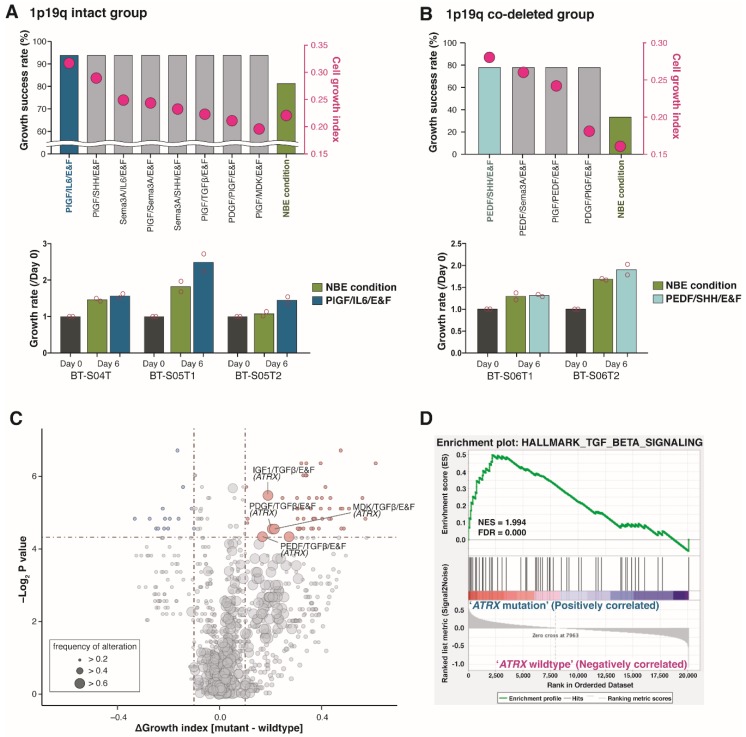
Newly selected culture conditions for *IDH1*-mut DIGs. (**A**,**B**) The culture conditions with higher growth success rates than the NBE condition for the *IDH1*-mut and 1p19q intact cells (**A**) and *IDH1*-mut and 1p1/9q co-deleted cell (**B**) (upper panels). The bars and dots indicate growth success rates and cell growth indices, respectively. The culture condition with the highest growth success rate and highest cell growth index was validated in additional samples (lower panels). The dots represent the values from technical duplicates. (**C**) A volcano plot showing culture conditions with significant differences in cell growth indices between *IDH1*-wt and -mut GSCs for frequently mutated genes in DIGs. The X-axis represents the magnitude (cell growth index of *IDH1*-mut GSCs–that of *IDH1*-wt GSCs), and the Y-axis represents the significance (−log10(Wilcoxon rank-sum *P*-value)) of the association between mutations and culture conditions. Each circle represents a single mutation-culture condition interaction, and the size is proportional to the mutation frequencies. (**D**) The GSEA plot of transforming growth factor-beta (TGF-β)-associated gene set (HALLMARK_TGF_BETA_SIGNALING) between *ATRX*-mut and -wt GSCs in *IDH1*-mut DIGs from the TCGA LGG dataset.

When the cell growth index in each condition was evaluated depending on the 1p19q co-deletion status in *IDH1*-mut GSCs (Figure 5A,B), 1p19q-intact IDH1-mut GSCs were the most responsive to placental growth factor (PlGF)/interleukin-6 (IL-6)/E&F, and showed 1.44-fold higher cell growth index than under NBE conditions (Figure 5A, upper panel). On the other hand, the PEDF/sonic hedgehog (SHH)/E&F condition was the most effective for promoting cell proliferation in 1p19q co-deleted *IDH1*-mut GSCs, and the cell growth index of this condition was 1.75-fold higher than that of NBE condition (Figure 5B, upper panel). The effectiveness of these newly identified culture conditions was validated in additional GSCs (Figure 5A,B, lower panels).

Furthermore, to improve culture conditions for *IDH1*-mut GSCs with specific mutations, we examined the association between the genomic mutations and cell growth indices of all growth factor combinations in *IDH1*-mut GSCs (Figure 5C). This revealed that *IDH1*-mut GSCs with *ATRX* mutations showed higher cell propagation levels in culture media, which included transforming growth factor-beta (TGF-β)-containing including ‘neurTGF-β/E&F’, ‘platelet-derived growth factor (PDGF)/TGF-β/E&F’, and ‘MDK/TGF-β/E&F’ conditions compared to *ATRX*-wt *IDH1*-mut GSCs (Figure 5C and Table S4); this finding is supported by the observation that a TGF-β-dependent pathway was significantly enriched in *IDH1*-mut DIGs with *ATRX* alterations compared to those without *ATRX* alterations (Figure 5D, NES = 1.994, false discovery rate < 0.001) in the TCGA LGG dataset. Moreover, astrocytomas are significantly enriched for the TGF-β-dependent pathway compared to oligodendrogliomas and normal tissues in the Repository for Molecular Brain Neoplasia Data (REMBRANDT) dataset as well (Figure S5).

## 3. Discussion

Owing to their relatively favorable prognosis compared to *IDH1*-wt glioblastomas, *IDH1*-mut glioblastomas and LGGs have not been extensively investigated [33]. However, all DIGs are characterized by extensive infiltrative growth, neovascularization and resistance to various combined therapies and, despite variable intervals, the remaining tumors after neurosurgical resection result in tumor evolution to a more refractory and aggressive form [3,4,5]. GSCs are critical therapeutic targets, because these cells are responsible for DIG progression and evolution [17]. The act of passaging cells itself has been shown to activate cellular plasticity, altering the gene expression and phenotype of patient-derived glioblastoma cells [14,34,35]. Time is also a critical component for the utility of our functional pipeline to inform and succeed in precision oncology. Therefore, short-term cultured patient-derived GSCs continue to be a preferred model as they retain the capacity to differentiate into multiple lineages and recapitulate the genetic/molecular/biological profiles of parental gliomas, offering a unique opportunity for streamlining drug testing and evaluating pathophysiology [9,10,14].

As current GSC culture methods have many limitations for various high-throughput applications due to limited supply, culture instability and genetic background variability, it is imperative to improve cell culture format based on concrete scientific rationales for the expansion and storage of a DIG patient-derived spheroid biobank. Slight changes in the concentration of various additive growth factors can alter cell behavior and drug sensitivity [35]. As culture conditions are a key factor limiting the efficacy of current in vitro models, improved GF combinations based on scientific rationale are needed for expanding and storing DIG patient-derived GSCs. Herein, we describe a precision approach that combines genomic characterization, patient-derived GSCs isolated from the diverse grades of DIGs, and a newly-constructed GFSCAN that is a fast, cost-effective, and reliable screening platform for overcoming the scarcity of translational research for diverse grades of DIGs with genetic background variability. We evaluated the 6-day cell growth rates of 72 GSCs against 132 distinct combinations, using 13 tumor-associated GFs in a 384-well GFSCAN plate. The 13 GFs, including E&F, MDK, PlGF, IL-6, PEDF, SHH, IGF1, TGF-β, neuregulin 1 (NRG-1), hepatocyte growth factor (HGF), PDGF and semaphorin 3A (Sema3A) have been linked to the aggressive phenotypes of gliomas, such as cell proliferation, survival, invasion, cancer stemness and the resistance to standard therapies [14,16,18,27,29,36,37,38,39,40,41,42,43,44,45,46,47,48,49,50,51,52,53,54,55,56,57,58,59,60,61,62,63,64,65,66,67,68,69,70,71,72,73,74,75,76,77,78,79,80,81,82,83,84].

As IDH is a component of the Krebs cycle and converts isocitrate and cofactor NAD^+^ to carbon dioxide, NADH and α-ketoglutarate; *IDH* mutations cause a buildup of the onco-metabolite D-2-hydroxyglutarate, resulting in the distinct expression of a GSC phenotype [22]. LGGs and glioblastomas within our cohort demonstrated distinct gene expression patterns and GSCs from glioblastomas were more dependent on E&F than LGG PDCs. Especially among glioblastomas, GSCs with an *IDH1* mutation were less dependent on E&F than those with wildtype *IDH1*, indicating that GSCs could show distinct gene expression patterns and dependency on E&F according to their *IDH1* mutation status. First, we identified PEDF/MDK/E&F as the best GF combination for mesenchymal *IDH1*-wt GSCs that are less dependent on E&F for cell growth and have relatively low cell-cycling activity. In contrast, most *IDH1*-wt GSCs with proneural subtype traits were dependent on E&F. Consistent with our findings, gliomas with molecular profiles comprised of a mesenchymal signature represent an invasive phenotype with a low proliferating activity. This phenotype is regulated by PI3K/AKT, nuclear factor-κB, TGF-β, MET, Wnt/β-catenin activation, and metabolic reprogramming of glycolysis by triggering the expression of epithelial to mesenchymal transition (EMT) activators [85]. Mechanistically, PEDF is involved in the self-renewal and maintenance of infiltrating GSCs as an autocrine factor by activating a signal transducer, an activator of transcription 3 (STAT3) and the Notch-Sox2 signaling axis [60]. Additionally, MDK, which is upregulated by Wnt/β-catenin in gliomas, contributes to tumor progression and metastasis by enhancing the growth, survival, and EMT of cancer cells; this is because MDK activates a Notch signaling that cross-talks with PI3K/AKT and STAT3 signaling [64,65,86,87]. These previous reports are consistent with the growth-promoting effects of MDK on E&F-independent *IDH1*-wt glioblastomas with high mesenchymal activity. 

The recent WHO 2016 criteria utilized 1p19q co-deletion to distinguish *IDH*-mutated DIGs with oligodendroglial phenotypes from astrocytomas [1,3,4]. We tried to figure out the best culture condition for glioblastoma with *IDH1* mutation and LGG with/without 1p19q co-deletion, previously known as hard to grow and easily eliminated in standard NBE culture conditions [24,26,30,31,32]. The differences in the culture success rate, depending on clinical and biological aggressive tumors, supported the difficulty in the generation of the GSCs from these subsets [10,24,26,88,89]. There is a compelling need for more reliable in vitro GSC libraries that mimic the biology of human LGGs and *IDH1*-mut glioblastomas in situ, which can be expanded to include considerable numbers of patients, and can facilitate the identification of drug sensitivity profiles for small subsets of patients with significant results. In this study, the PEDF/SHH/E&F combination was critical for the steady propagation of a subset of *IDH1*-mut GSCs harboring 1p19q co-deletion and PlGF/IL-6/E&F was essential for another subset of *IDH1*-mut GSC spheroids with intact 1p19q. On the other hand, *ATRX* mutations are mutually exclusive from 1p19q co-deletion, but are associated with *IDH1* mutation, suggesting that ATRX drives the lineage-specific formation of astrocytomas [1,3,4]. Among all the 132 conditions, three containing TGF-β (IGF1/TGF-β/E&F, PDGF/TGF-β/E&F, and MDK/TGF-β/E&F) were particularly effective for cultivating *IDH1*-mut GSCs harboring *ATRX* mutations compared to *ATRX*-wt GSCs. Interestingly, we also confirmed that TGF-β signaling was up-regulated in *ATRX*-mut DIGs or astrocytomas in the independent public dataset, highlighting the tumor-promoting role of TGF-β in DIGs with *ATRX* mutations.

In summary, we have identified the optimal culture conditions for propagating GSCs, according to their major genetic alterations in spheroid culture, using large-scale GF screens. Our versatile GFSCAN tool could figure out the optimal culture conditions for other cancer types, for which robust and reliable in vitro culture conditions need to be determined. Most importantly, the complex cross-talk between a variety of signaling pathways associated with GSCs support our findings that ‘PDGF/IL6/E&F’, ‘PDGF/PlGF/E&F’ and ‘PEDF/SHH/E&F’ could be alternative supplements to standard E&F alone, by their synergistic promoting effects on the establishment of GSCs from *IDH1*-mut glioblastomas and LGGs. These newly identified GFs do not function alone, but rather their combinations generate complex and dynamic interactions that can synergistically promote the propagation of patient-derived GSCs in vitro. The findings from the present study suggest that the mechanisms underlying the association between specific genomic alterations and specific GF combinations, and the more in-depth elucidation of biomarkers tailoring optimal culture media, should be further explored using a larger panel of GSCs and machine learning. Additional GFs and combinations should also be tested and validated in external GSC sample sets from DIGs in prospective cohort studies. 

## 4. Materials and Methods 

### 4.1. Preparation of Patient-Derived GSCs from Human DIGs 

Fresh tumor specimens and corresponding clinical records were obtained from 60 patients with DIGs (Figure 2 and Table S2) who underwent surgery at the Samsung Medical Center (Seoul, Korea), in accordance with the guidelines of the institutional review board (No. 2005-05-001, 2010-04-004). Informed consent was obtained from each patient prior to the study. Portions of surgical samples were enzymatically dissociated into single cells using Liberase^TM^ (Roche Diagnostics, Basel, Switzerland) and red blood cells were removed by density gradient centrifugation with Percoll for GFSCAN and stored at −80 °C for further genomic characterization and GFSCAN. The dissociated cells were centrifuged for 3 minutes at 1200 rpm after counting and the cell pellet was gently suspended with 1 mL of CELLBANKER2 (a serum-free cell cryopreservation medium with a unique complete formulation for the broad spectrum of mammalian cell cultures, ZENOAQ, #BLC-2) cryopreservation medium by 1–2×10^6^ cells per vial on average. The vial was stored in freezing tank, frozen slowly at −80 °C for one day and then stored in a liquid nitrogen tank. For cell thawing, we devised a new process, which requires no growth factors and improves early cell viability (Figure S6); Cells were thawed at 37 °C and placed slowly in a culture flask containing pre-warmed (37 °C) media, so that the dissolved cells contained less than 1% of DMSO in the culture flask. The flask was incubated at 37 °C for 4 h and then cells were centrifuged. The centrifuged cell pellet was re-suspended in fresh media and incubated at 37 °C for a day. We compared the 1-day cell viability of the new cell thawing method with conventional thawing and confirmed that the new method resulted in a higher 1-day cell viability (Figure S6).

### 4.2. Manufacturing GFSCAN Based on Patient-Derived GSCs

We established a novel screening platform to identify advanced in vitro culture conditions by systematically evaluating the pro-survival and pro-proliferation effects of various oncogenic GFs on GSCs. The frozen dissociated cells were thawed and seeded into 384-well plates (1000 cells in 50 μL media/well) in technical duplicates. The cells were cultured in the GFSCAN plate for 6 days and cell growth was measured using ATPlite^TM^ (PerkinElmer, Waltham, MA, USA) (Figure 1). First, we selected 11 growth factors previously reported to enhance cell survival, proliferation, cancer stemness, and invasion, particularly in malignant glioma, in addition to E&F. These included IGF1, NRG-1, HGF, PDGF, PlGF, PEDF, Sema3A, TGF-β, IL-6, SHH, and MDK. GSCs were cultured in neurobasal medium with N2 and B27 supplements (0.5× each; Gibco, Grand Island, NY, USA) and various combinations of 11 GFs and E&F (5 ng/mL EGF; 5 mg/mL bFGF; 1 ng/mL IGF1; 1 ng/mL NRG-1; 1 ng/mL HGF; 1 ng/mL PEDF; 1 ng/mL PDGF; 1 ng/mL PlGF; 1 ng/mL SHH; 1 ng/mL Sema3A; 1 ng/mL TGF-β; 1 ng/mL IL-6; 1 ng/mL MDK). Detailed information of growth factors tested in the present study is summarized in Table S1. Except for E&F, two of the 11 growth factors were selected and mixed to form 55 combinations. These 11 growth factors were also used alone under 11 single conditions. As the E&F combination is the basic condition required for GSC culture, E&F were added to the 55 combinations (culture conditions with E&F), while another 55 combinations did not contain E&F (culture conditions without E&F). GFSCAN assays containing these 132 conditions and two control conditions (no growth factor and NBE) were set up by the JANUS ‘cherry-picking system’ (PerkinElmer). A schematic workflow is summarized in Figure 1.

### 4.3. GFSCAN QC Criteria

The ATPlite^TM^-based cell viability data obtained from the GFSCAN platform were analyzed only when they met the following quality control criteria: 1) the ATPlite^TM^ value must be more than 5000, as we assume that a sinal intensity lower than 5000 indicates that the number of viable tumor cells are very low and negligible and 2) the ‘6 day/0 day’ ratio must be more than 0.5. 

### 4.4. Genomic and Transcriptomic Analysis 

For all 72 DIG samples (19 LGGs and 53 glioblastomas from 60 patients; summarized in Table S2) that were subjected to GFSCAN, either WES (n = 35) or glioma-specific targeted DNA sequencing (GliomaSCAN^TM^, n = 37) was performed to reveal single-nucleotide variations, insertions and deletions, and copy number alterations (CNAs) (Tables S2 and S3). Data from WTS were also obtained to determine the gene expression profiles and structural variants of the 72 DIGs. For WTS, total RNA was extracted from patient-derived cells and tumor tissues using the RNeasy Mini Kit (Qiagen, Hilden, Germany), according to the manufacturer’s instructions. A 2100 Bioanalyzer (Agilent Technologies, Santa Clara, CA, USA) was used to assess RNA integrity. RNA-Seq libraries were prepared using the TruSeq RNA Library Prep kit (Illumina, San Diego, CA, USA). From the DNA and RNA sequencing data, mutation calling, CNA estimation, exon skipping detection, and gene expression profiling were processed using the methods reported in our previous publication [90]. 

The gene expression levels were summarized in reads per kilobase million (RPKM) values, and the log2-transformed (RPKM + 1) values were used for further analyses. Moreover, although high levels of similarity in mutations and gene expression patterns have been reported between parental tumor tissues and matched patient-derived GSCs [14], we selected genes showing highly positive correlations (Spearman correlation coefficient values ≥ 0.4) between GSCs and their parental tissues using our in-house DIG WTS dataset. Then, we removed unannotated genes and the genes with maximum log2(RPKM + 1) values = 0 in more than half of tumor tissues or half of GSCs. Finally, 3208 genes were left and assumed to be tumor-intrinsic genes, and we used only these genes for further analysis due to the lack of microenvironment-specific genes in GSCs, with the exception of GSEA analysis and subtype assignment (Figure S7). DEGs between E&F-dependent and -independent samples were extracted by DEGseq (*p* value ≤ 0.05 and Benjamini q value ≤ 0.1) [91]. These DEGs were subjected to the Nearset Template Prediction (NTP) algorithms (R package, NTPez) to classify additional DIG samples with RNA sequencing into either E&F-dependent or -independent groups [92].

### 4.5. Unsupervised Hierarchical Clustering of Cell Growth Index from GFSCAN

Based on cell growth indices of 72 DIGs, unsupervised hierarchical clustering was performed in R with Ward linkage and Euclidean distance methods. 

### 4.6. Pathway Enrichment Analysis and GSEA

GSEA was conducted using the GenePattern software of the Broad Institute (http://software.broadinstitute.org/cancer/software/genepattern) [93]. The statistics to measure gene set enrichment were estimated by 1000 gene set permutations.

### 4.7. Glioblastoma Molecular Subtype Classification

To determine the molecular subtype of *IDH1*-wt GSCs based on their gene expression profiles, R package GSVA (gene set variation analysis) was used to calculate single-sample GSEA (ssGSEA) scores for each subtype (three glioblastoma-intrinsic subtypes) of GSCs [28,94]. Then, ssGSEA scores were normalized across the GSCs, and the subtype with the highest normalized score was assigned to each sample. 

### 4.8. External Gene Expression Data Sets 

Gene expression data and *ATRX* mutation status from TCGA LGG dataset were downloaded from the Genomic Data Commons (GDC) (https://portal.gdc.cancer.gov/) using the R package TCGAbiolinks [95]. Microarray raw data (CEL files) and clinical information from the Repository of Molecular Brain Neoplasia Data (REMBRANDT) were downloaded and processed from the REMBRANDT data portal in 2011 (now available in ArrayExpress E-MTAB-207) [96].

### 4.9. Statistical Analysis

GraphPad Prism software was used for all statistical analyses (GraphPad, Inc., La Jolla, CA, USA). Column statistics were used to display the differences in growth rate between each group. Data are expressed as the mean ± standard error of mean (SEM), two-tailed *t*-tests of variance were performed whenever appropriate and *p* values < 0.05 were considered as significant. Spearman rank correlation coefficients were also calculated. Spearman’s correlation coefficients and significance (two-tailed) were calculated for each pair of genes. 

## 5. Conclusions

In this study, we successfully established a robust system called GFSCAN that evaluates 6-day cell growth rates under 132 culture conditions. Using GFSCAN, we evaluated the cell growth indices of 72 DIG patient-derived cells and also obtained their matched genomic and transcriptomic profiles to explore the association between cell growth and genomic characteristics. Finally, it was found that mesenchymal *IDH*-wt glioblastomas were less proliferative under current standard culture conditions compared to proneural and classical glioblastomas, and that the PDEF/MDK/E&F combination could be an alternative to NBE for improving the propagation ability of mesenchymal GSCs. *IDH1*-mutant GSCs also showed significantly lower cell growth indexes than *IDH1*-wt GSCs under NBE. Therefore, we identified new GF combinations for *IDH1*-mut GSCs. Furthermore, PIGF/IL-6/E&F and PEDF/SHH/E&F effectively increased cell growth indexes for *IDH1*-mut/1p19q intact GSCs and *IDH1*-mut/1p19q co-deleted GSCs, respectively. Moreover, TGF-β-containing culture conditions with E&F were shown to promote the growth of *IDH1*/*ATRX*-mut GSCs.

## 6. Patents

A patent covering GFSCAN has been filed under publication number WO2019050314A1.

## Supplementary Materials

The following are available online at https://www.mdpi.com/2072-6694/12/3/549/s1, Figure S1: Validation of GFSCAN reproducibility via biological duplicates for each glioblastoma sample (BT-S07T (left panel) and BT-S08T (right panel)), GSCs from two independent frozen vials were thawed, and GFSCAN was performed twice (1st vial: 1st experiment and 2nd vial: 2nd experiment), Figure S2: Distinct genetic and biologic characteristics of glioblastomas and LGGs, Figure S3: Treatment with midkine (MDK)-neutralizing antibody inhibits cell proliferation, Figure S4: Classification of additional IDH1-wt glioblastoma samples into E&F-dependent and -independent groups using gene expression surrogate markers, Figure S5: GSEA enrichment plot of HALLMARK_TGF_BETA_SIGNALING in REMBRANDT dataset, Figure S6. The new cell thawing method minimizes cell damage, Figure S7: Selection of 3208 tumor-specific genes in DIGs, Table S1: Product information for growth factors used in GFSCAN, Table S2: Sample information of 72 screening and 8 validation DIGs, Table S3: Single nucleotide variant (SNV) and insertion/deletion (INDEL) list of DIG samples based on GliomaSCANTM (GS) and whole exome sequencing (WES), Table S4: Cell growth index under all 133 conditions including NBE (E&F) condition, Table S5: DEGs between E&F-dependent and -independent IDH1 wt GSCs in GFSCAN cohort.
