# Supplementary material for: Sphere-Forming Culture for Expanding Genetically Distinct Patient-Derived Glioma Stem Cells by Cellular Growth Rate Screening
Source: Cancers (Basel). 2020 Feb 27;12(3):549. doi: 10.3390/cancers12030549 (PMC7139415; doi:10.3390/cancers12030549)
Supplement: Supplementary file 1 [file cancers-12-00549-s001.zip › Cancers-663915-supplementary Figures.docx]

Supplementary Figures:

Sphere-Forming Culture for Expanding Genetically Distinct Patient-Derived Glioma Stem Cells by Cellular Growth Rate Screening

Kayoung Shin ^†^, Hyemi Shin ^†^, Hee Jin Cho ^†^, Hyunju Kang, Jin-Ku Lee, Yun Jee Seo, Yong Jae Shin, Donggeon Kim, Harim Koo, Doo-Sik Kong, Ho Jun Seol, Jung-Il Lee, Hye Won Lee * and Do-Hyun Nam *


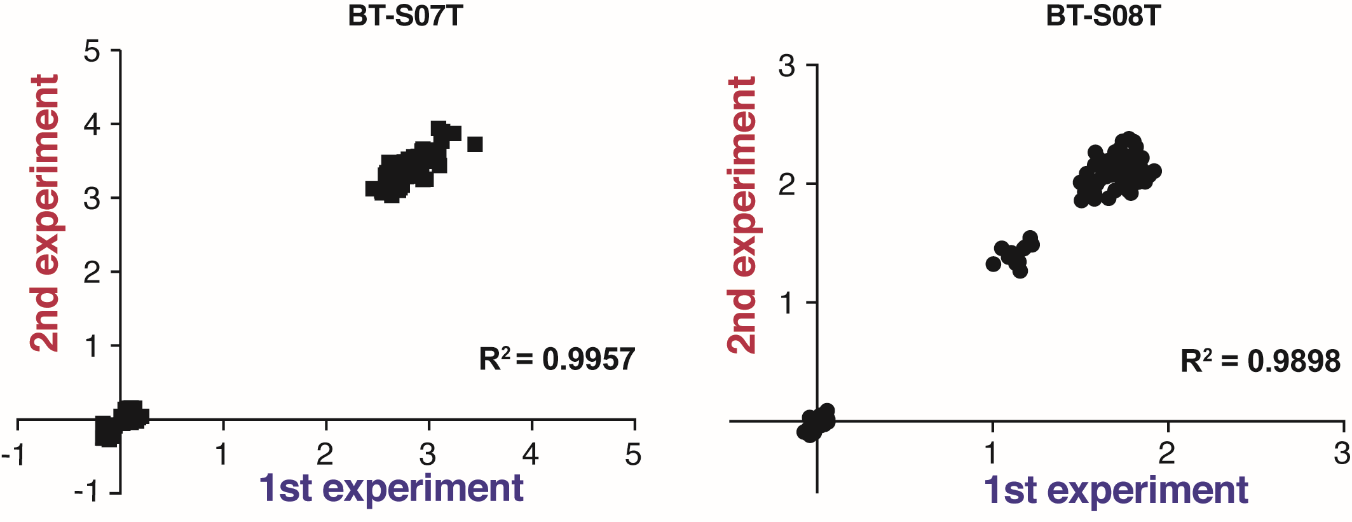


**Figure S1.** Validation of GFSCAN reproducibility via biological duplicates for each glioblastoma sample (BT-S07T (left panel) and BT-S08T (right panel)), GSCs from two independent frozen vials were thawed, and GFSCAN was performed twice (1st vial: 1st experiment and 2nd vial: 2nd experiment). Each dot represents the intensity from two repeated experiments under 132 growth factor conditions. Both cases showed strong positive correlation between the two experiments (R^2^ = 0.9957 and 9898), representing GFSCAN reproducibility.


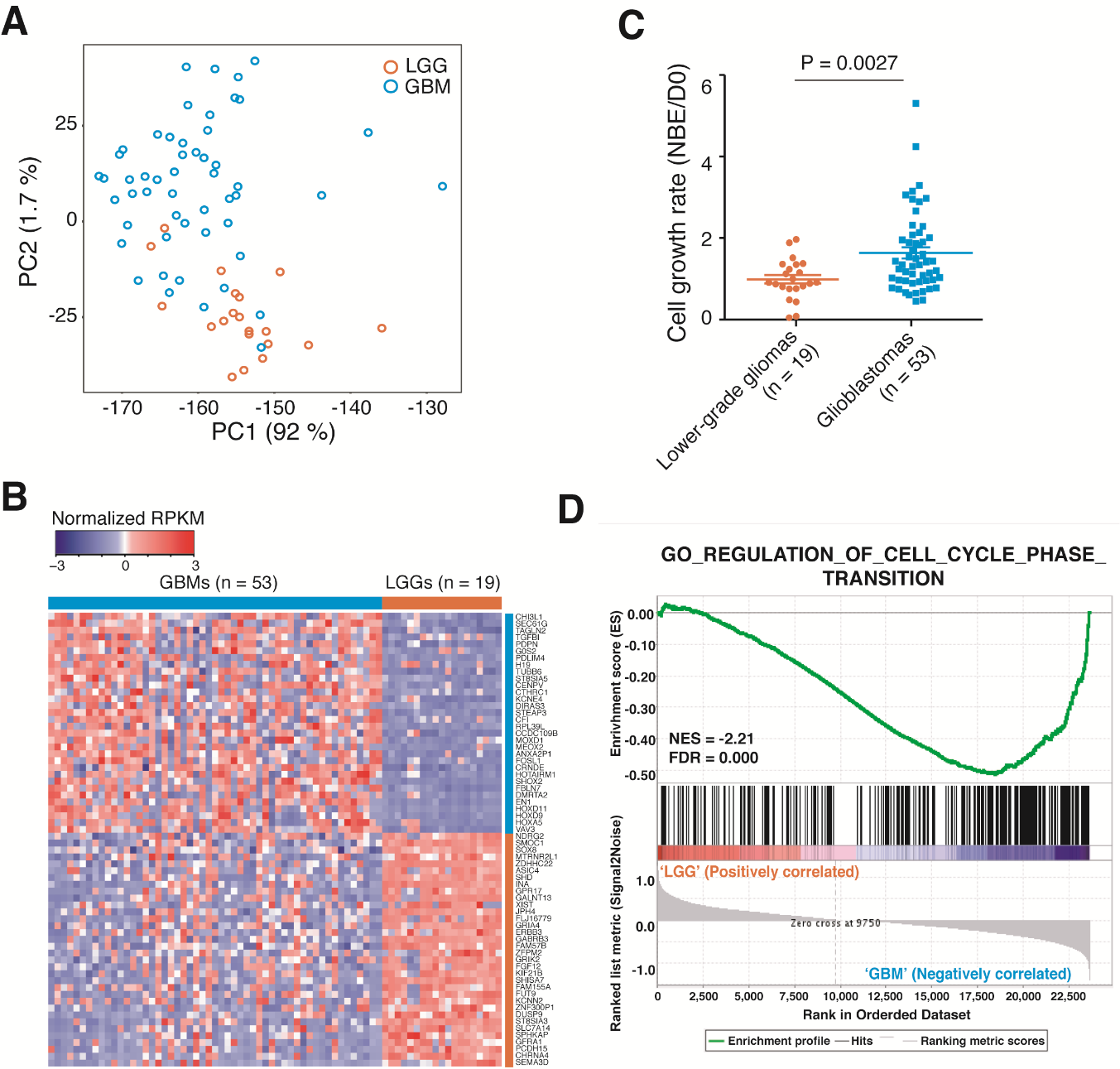


**Figure S2.** Distinct genetic and biologic characteristics of glioblastomas and LGGs. **(A)** Principle component analysis using tumor-intrinsic gene expression profiles of 72 GFSCAN DIGs. Blue and orange dots indicate glioblastoma and LGG GSCs, respectively. **(B)** Genes that are differentially expressed between GFSCAN glioblastoma and LGG GSCs (*p* value ≤ 0.05 and Benjamini q value ≤ 0.1). **(C)** 6-day growth rates of GSCs form LGGs and glioblastomas under no growth factor condition (left panel, *p* = 0.134) and NBE (right panel, *p* = 0.0027). **(D)** GSEA plot of a cell cycle-associated pathway (GO_ REGULATION_OF_CELL_CYCLE_PHASE_TRANSITION) between glioblastoma and LGG GSCs.


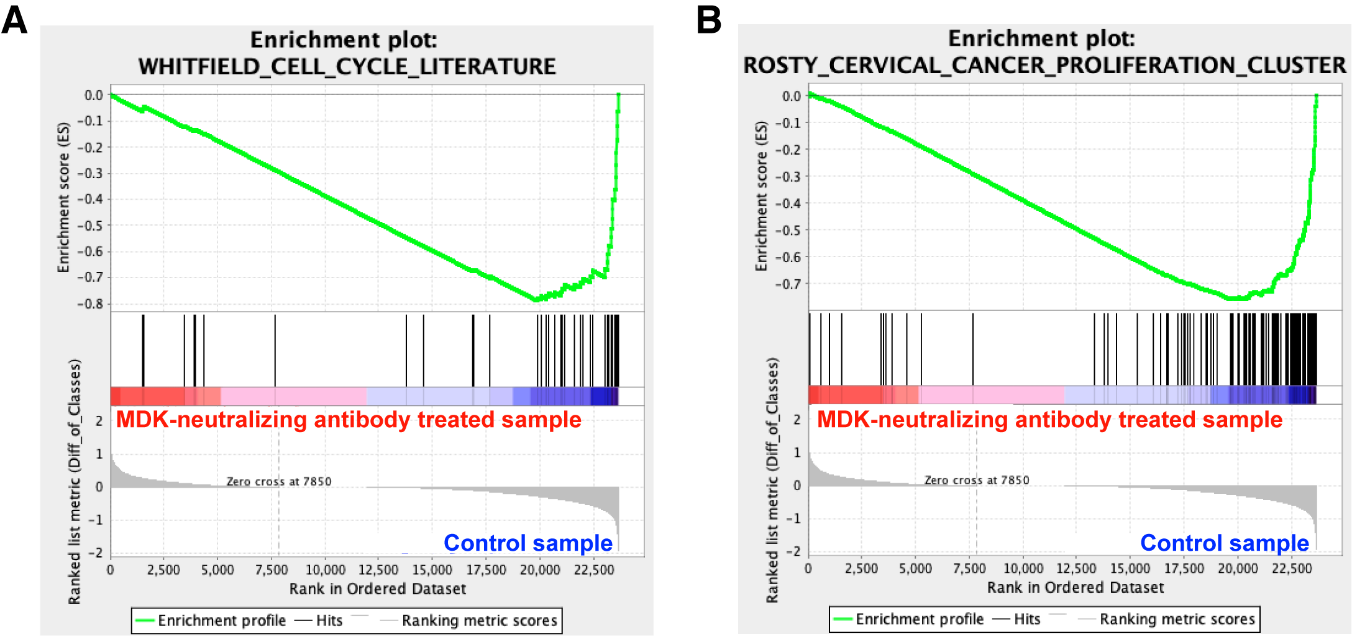


**Figure S3.** Treatment with midkine (MDK)-neutralizing antibody inhibits cell proliferation. **(A,B)** GSEA plot of a cell proliferation (WHITFIELD_CELL_CYCLE_LITERATURE **(A)** and ROSTY_CERVICAL_CANCER_PROLIFERATION_CLUSTER **(B)** between MDK-neutralizing antibody treated sample and non-treated sample.


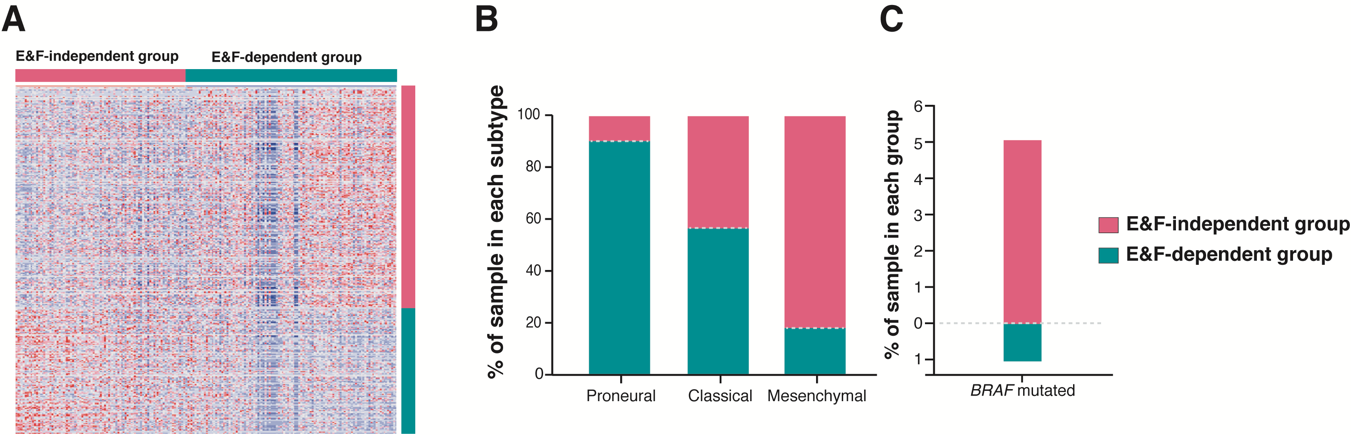


**Figure S4.** Classification of additional *IDH1*-wt glioblastoma samples into E&F-dependent and -independent groups using gene expression surrogate markers. **(A)** Nearest template prediction was performed with 202 validation samples (*IDH1*-wt glioblastomas) that were not included in the screening set. **(B)** Ratio of E&F-dependent and -independent samples in each glioblastoma subtype in validation cohort. **(C)** Ratio of samples with *BRAF* hotspot mutations in E&F-dependent and -independent group in validation cohort.


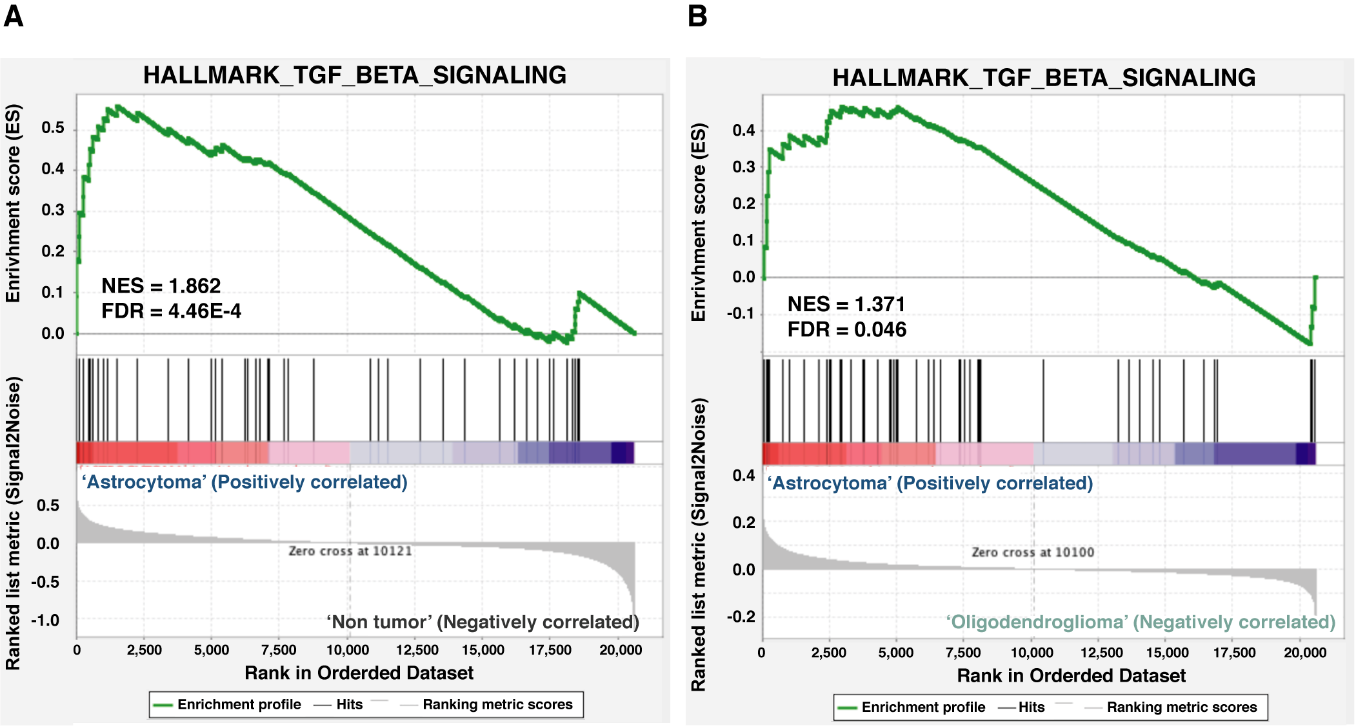


**Figure S5.** GSEA enrichment plot of HALLMARK_TGF_BETA_SIGNALING in REMBRANDT dataset. **(A,B)** TGF-β associated gene set was significantly enriched in astrocytoma compared to non-tumor **(A)** and oligodendroglioma **(B)**.


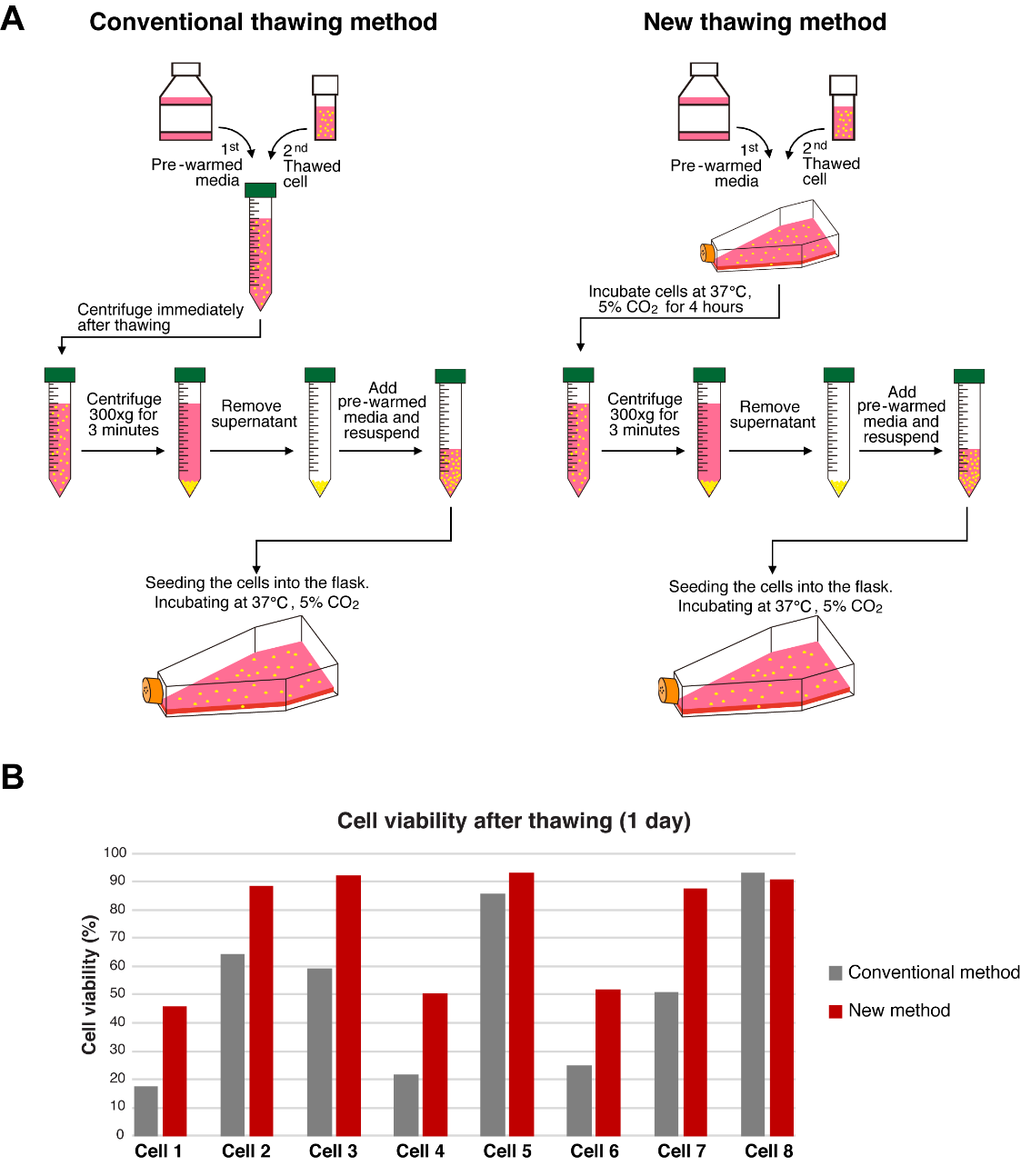


**Figure S6.** The new cell thawing method minimizes cell damage. **(A)** Differentiated from the traditional method, a protocol for incubating cells for 4 hours immediately after thawing was applied **(B)** Compared with the previous method, the viability of 7 of 8 cell lines 1 day after thawing was higher when applying the new method.


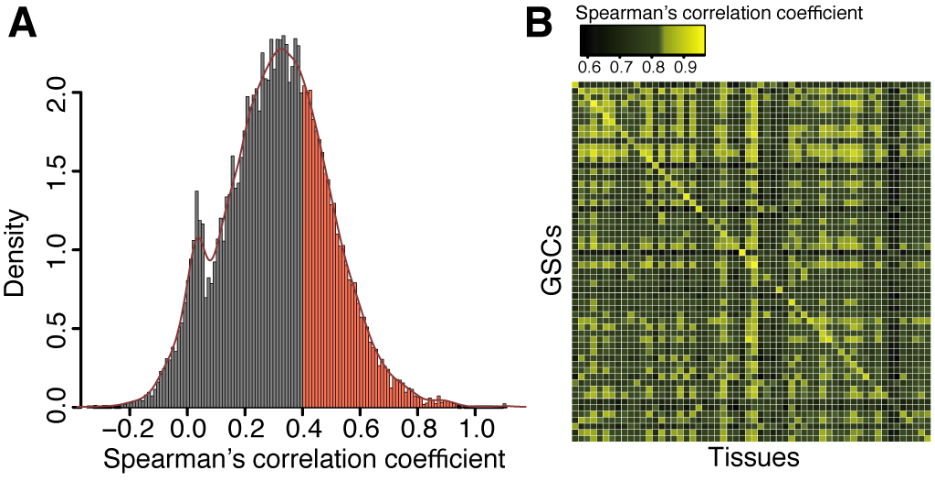


**Figure S7.** Selection of 3,208 tumor-specific genes in DIGs. **(A)** Distribution of Spearman correlation of whole genes between GSCs and their parental tissues. Red bars indicate Spearman rho values > 0.4, and 3,208 genes that belong to red bars are used for principal component analysis and DEG analysis as tumor-intrinsic genes. **(B)** A heatmap showing Spearman correlations between GSCs and their parental tissues. Each pair of GSC and parental tissue shows a high Spearman correlation rho value.
